# A Key Role for Membrane Transporter NKCC1 in Mediating Chondrocyte Volume Increase in the Mammalian Growth Plate

**DOI:** 10.1002/jbmr.47

**Published:** 2010-01-29

**Authors:** Peter G Bush, Meredith Pritchard, Mohamad Y Loqman, Timothy A Damron, Andrew C Hall

**Affiliations:** 1 Centre for Biomedical and Health Science Research, School of Pharmacy and Biomolecular Sciences, University of BrightonBrighton, UK; 2 Musculoskeletal Sciences Research Laboratory, Department of Orthopedic Surgery, State University of New York Upstate Medical UniversitySyracuse, NY, USA; 3 Centre for Integrative Physiology, School of Biomedical Sciences, University of EdinburghEdinburgh, Scotland, United Kingdom

**Keywords:** growth plate, hypertrophic chondrocytes, NKCC, volume, rat

## Abstract

The mechanisms that underlie growth plate chondrocyte volume increase and hence bone lengthening are poorly understood. Many cell types activate the Na-K-Cl cotransporter (NKCC) to bring about volume increase. We hypothesised that NKCC may be responsible for the volume expansion of hypertrophic chondrocytes. Metatarsals/metacarpals from 16 rat pups (P_7_) were incubated in the presence/absence of the specific NKCC inhibitor bumetanide and measurement of whole-bone lengths and histologic analysis of the growth plate were done after 24 hours. Fluorescent NKCC immunohistochemistry was visualised using a confocal laser scanning microscopy on seven rat tibial growth plates (P_7_). Microarray analysis was performed on mRNA isolated from proliferative and hypertrophic zone cells of tibial growth plates from five rats of each of three ages (P_49/53/58_). Exposure to bumetanide resulted in approximately 35% reduction (paired Student's *t* test, *p* < .05) of bone growth in a dose-dependent manner; histologic analysis showed that a reduction in hypertrophic zone height was responsible. Quantification of fluorescence immunohistochemistry revealed a significant (paired Student's *t* test, *p* < .05) change in NKCC from the intracellular space of proliferative cells to the cytosolic membrane of hypertrophic zone cells. Further, microarray analysis illustrated an increase in *NKCC1* mRNA between proliferative and hypertrophic cells. The increase in *NKCC1* mRNA in hypertrophic zone cells, its cellular localization, and reduced bone growth in the presence of the NKCC inhibitor bumetanide implicate NKCC in growth plate hypertrophic chondrocyte volume increase. Further investigation is warranted to determine the regulatory control of NKCC in the mammalian growth plate and the possible detrimental effect on bone growth with chronic exposure to loop diuretics. © 2010 American Society for Bone and Mineral Research.

## Introduction

The growth plate is comprised of a thin layer of columnar chondrocytes lying perpendicular to and responsible for new bone formation during longitudinal skeletal growth. Cells within the column are highly organized, with a “reserve” zone preceding proliferative zone chondrocytes (PZCs). After a regulated period of time, a marked differentiation occurs, signified by a dramatic increase in volume, resulting in the formation of hypertrophic zone chondrocytes (HZCs). This volume increase is predominantly in the longitudinal axis, which accounts for approximately 80% of bone lengthening.(1) The volume of a typical cell increases from approximately 1000 µm^3^ in the PZC to approximately 15,000 µm^3^ in the HZC.(2) It has been postulated that this cell volume increase occurs through a combination of classic “hypertrophy”(2) and “swelling.”(3) However, the mechanisms that drive growth plate HZC volume expansion are unknown.

Cell swelling describes the process arising from the net movement of water into the cell, and in biologic systems, this process relies solely on an osmotic gradient. This can result from a reduction in extracellular osmolarity, an increase in intracellular osmolarity, or a combination of the two. Studies measuring the effect of hypoosmotic challenge on in situ growth plate chondrocytes suggest that for cell swelling to be entirely mediated by a reduction in extracellular osmolarity, these cells would need to be exposed to an approximately 280 mOsmol reduction in osmolarity(2) to approximately 120 mOsmol. Plasma osmolarity therefore would need to be reduced from approximately 300 mOsmol to near 20 mOsmol, which is clearly implausible. Therefore, osmotic gradient–driven water accumulation is more likely to be due to an *increase* in intracellular osmolarity in the transition of a PZC to an HZC.

Intracellular osmolarity can be raised by the catabolism of intracellular macromolecules (eg, proteins and/or complex carbohydrates) into osmotically active components (eg, amino acids and/or simple sugars).(4) However, intracellular organic osmolytes do not account for the volume increase between PZC and HZC.(5) Therefore, the “active” movement of osmolytes across the plasma membrane into the cell is the most probable mechanism. Previous studies suggest that the accumulation of organic solutes (eg, simple sugars and amino acids) is responsible for approximately 9% of the osmolytes required.(5) This strongly suggests other osmolytes are involved, and the activity and/or number of inorganic solute transporters also may be increased in order for cells to amass appropriate quantities of osmolytes to drive such a volume increase.

The intracellular sodium concentration ([Na^+^]_*i*_) plays a key role in determining cell volume, and Na^+^ is moved across the cytoplasmic membrane through a range of transport proteins (see ref. (6)). One of these, the Na-K-2Cl cotransporter (NKCC), is an obvious candidate for HZC expansion owing to its ability to increase cell volume in other cell types.(7) NKCC mediates electroneutral ion transport and is characterized by its sensitivity to loop diuretics (eg, bumetanide and furosemide).(8) In many cell types, activation of NKCC (eg, by exposure to hypertonicity) results in an increase in cell volume, termed *regulatory volume increase* (RVI).(6,7) Typically, RVI describes volume recovery after cell shrinkage, returning a cell to its “normal” volume. In contrast, growth plate chondrocytes require a coordinated, continual volume increase as they progress from PZC to terminal HZC phenotypes. Increased activity of NKCC may be able to drive such cell swelling.

Two NKCC isoforms are known, the near-ubiquitous NKCC1 and NKCC2, which appears to be localized exclusively in the kidney.(7–10) Although considered to be present in all tissues, to our knowledge, only mRNA for *NKCC1* has been reported in growth plates, where it demonstrates increased expression from PZCs to HZCs.(11)

In this study, we tested the hypothesis that NKCC1 was involved with HZC volume increase and longitudinal bone growth using a variety of methodologies. First, whole-metatarsal/metacarpal rudiments were incubated with the loop diuretic bumetanide, a specific NKCC inhibitor, which inhibited bone elongation by approximately 35%. Histologic analysis of growth plates from bumetanide-exposed metatarsal rudiments showed a reduction in HZ height, suggesting an inhibition of NKCC-mediated HZC volume increase. For bumetanide to be acting on NKCC in the hypertrophic zone, NKCC has to be associated with the HZC plasma membrane. To elucidate NKCC tissue and cellular distribution, immunohistochemistry was performed on growth plate sections. While NKCC immunofluorescence was present throughout the growth plate, it was most prevalent in HZCs. Further examination of NKCC cellular distribution revealed a dramatic change from a predominantly intracellular location in PZCs to the cytoplasmic membrane in HZCs. NKCC1-specific antibodies are not available, but gene array microanalysis confirmed the upregulation of *NKCC1* mRNA, with negligible signal for *NKCC2* mRNA.

## Materials and Methods

### Biochemicals and solutions

Unless otherwise stated, all biochemicals and solutions were obtained from Sigma Chemical Company (Poole, UK). Bone rudiment dissection media consisted of phosphate-buffered saline (PBS) containing α-medium (7.5% v/v; Invitrogen, Ltd., Paisey, UK) and bovine serum albumin V (1 mM). Standard culture medium (α-medium) was supplemented with Na_2_ glycerol biphosphate (1 mM), bovine serum albumin V (1 mM), and l-ascorbic acid (5 mg/mL). Bumetanide was prepared as a 20 mM stock solution in ethanol. 4,4'-Diisothiocyanatostilbene-2,2'-disulfonic acid (DIDS) was prepared as a 0.1 M stock solution in 0.1 M potassium bicarbonate.

### Animal preparation

For immunohistochemistry and bone rudiment culture, 19 Sprague-Dawley rat pups (7 days old, P_7_) were humanely killed by decapitation following UK Home Office guidelines for other experiments. The three middle metatarsals of each hind limb, three middle metacarpals from 14 of the animals, and tibias from 6 animals were dissected out while immersed in dissection medium and carefully cleaned of soft tissue.

Fifteen Sprague-Dawley rats were also used, aged 49, 53, and 58 days old (5 animals in each set). These older animals with larger growth plates were used to obtain sufficient material for microarray analysis studies. Animals were killed by carbon dioxide asphyxiation following procedures reviewed and approved by the Institutional Use and Care of Animals Committee. For the purpose of this study, the left proximal tibial bone including the growth plate then was harvested and immediately frozen in liquid nitrogen and stored at −80°C.

### Whole-metatarsal rudiment preparation and measurement

**Fig. 1:**
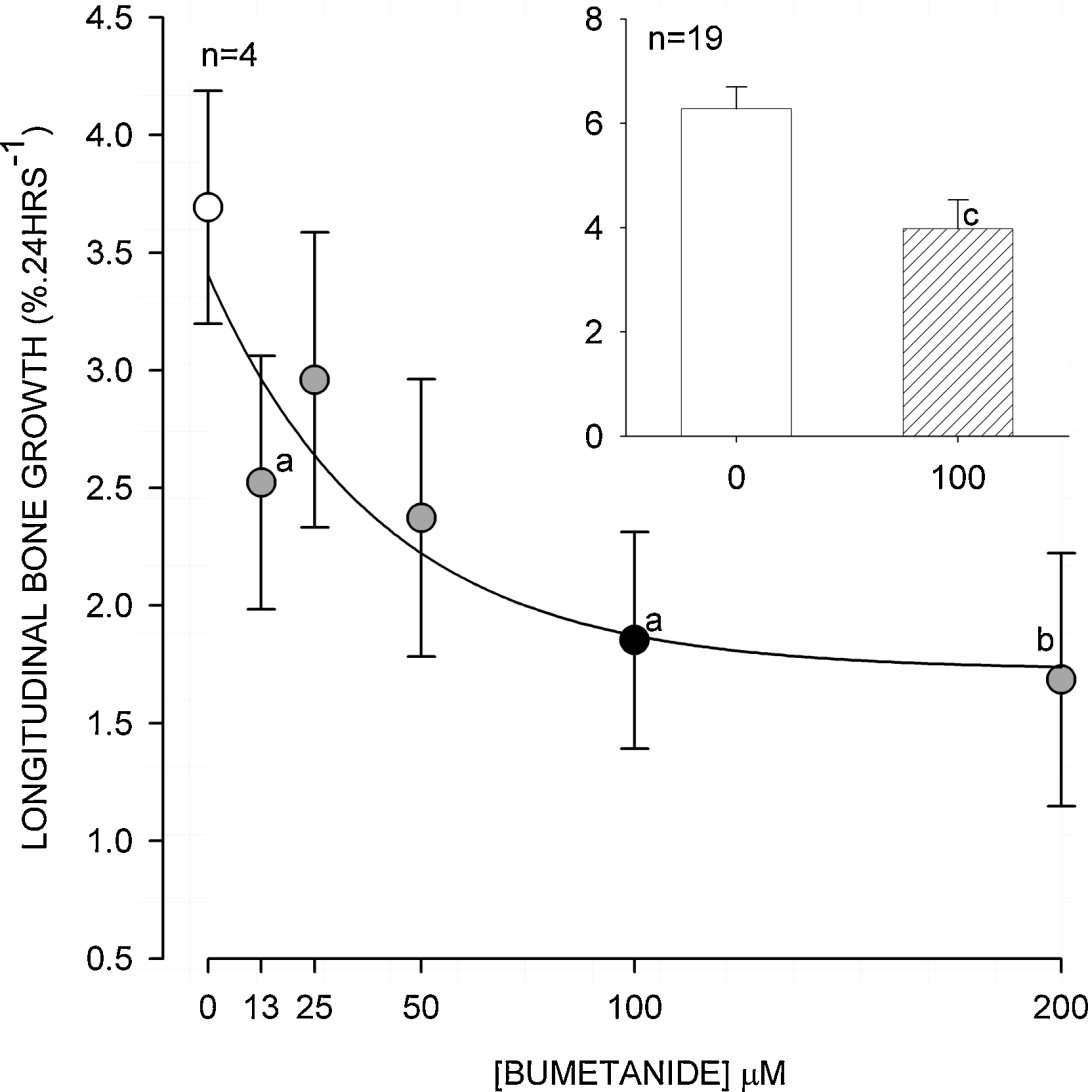
The effect of NKCC inhibition on rat metatarsal bone rudiment growth by the NKCC inhibitor bumetanide. Each data point contains paired metatarsals, and metacarpals from each animal (*main graph*) or metatarsals alone (*inset*) were cultured for 24 hours and measured as described in “Materials and Methods.” There was a dose-dependent relationship for bumetanide inhibition; individual points were significantly different from control (no bumetanide, *open circle*) with bumetanide concentrations of 12.5, 100, and 200 µM (^a^*p* < .05; ^b^*p* < .01, paired *t* test). The inset chart shows the significant reduction (^c^*p* < .002, paired *t* test) of bone lengthening by 100 µM of bumetanide (*pattern fill*) on metatarsals alone (*n* = 19). Data expressed as means with errors bars representing SEM.

In order to prepare duplicate samples for determination of the effects of six bumetanide concentrations (0 to 200 µM) on bone lengthening, metatarsals and metacarpals of four animals were used (12 bones per animal). Each duplicate consisted of one metatarsal and one metacarpal. Bones were cultured individually in 1 mL of standard culture medium and maintained at 37°C [CO_2_ (5%):air (95%), pH 7.4]. Rudiment pairs were randomly selected for either bumetanide (12.5 to 200 µM) or vehicle control (ethanol alone) addition to the medium. Owing to the slower growth rate of metacarpals, further experiments were performed (100 µM of bumetanide or vehicle controls) on metatarsals alone from a further 15 animals. A bumetanide concentration of 100 µM was chosen because it resulted in maximal inhibition of bone lengthening (Fig. 1), but it was still below the concentration known to disrupt the activity of other membrane-transport proteins.(12–14) Similarly, metatarsals and metacarpals were dissected from 10 animals and randomly selected for a range of DIDS concentrations (0 to 1 mM) in the presence of 100 µM of bumetanide. A minimum of four animals was used for each DIDS concentration.

Images (640 × 320 pixels) of rudiments were acquired immediately after dissection and after 24 hours in the presence/absence of inhibitor using an eyepiece camera (DCM35, Brunel Microscopes, Chippenham, UK) fitted to a dissecting stereomicroscope (Wild M3, Heerbrugg, Switzerland). An image of a rule was acquired to provide calibration, and bone lengths were measured using ImageJ (National Institute of Health, Bethesda, MD, USA).

**Fig. 2:**
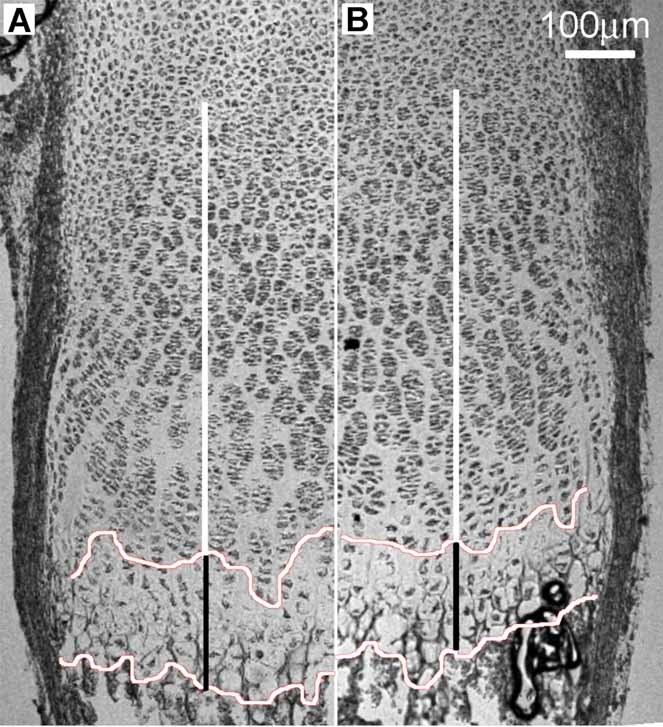
Light micrograph of rat metatarsal growth plate in the absence (*A*) and presence (*B*) of 100 µM of bumetanide. The irregular horizontal lines were traced by eye along the beginning of late HZCs that exhibited an unambiguous volume enlargement and the bottom at the zone of calcification. The black-and-white vertical bars indicate the average height of the late hypertrophic and remaining growth plate, respectively. Scale bar = 100 µm.

After 24 hours of incubation in the presence/absence of bumetanide, metatarsals were fixed with 4% paraformaldehyde in 0.1 M phosphate buffer, pH 7.4, overnight. After dehydration through a graded series of ethanol solutions, tibias were embedded in paraffin wax, cut into longitudinal 10 µm serial sections (Reichert-Jung 1130/Biocut microtome, Leica Biosystems Nussloch GmbH, Nussloch, Germany), and mounted onto polylysine-coated glass slides. Section wax was dissolved in xylene, followed by rehydration through a series of ethanol grades to PBS. Images of both proximal and distal growth plates were taken using the transmitted light detector of an upright confocal laser scanning microscope (Zeiss LSM510, Welwyn Garden City, UK) fitted with a ×10 dry objective lens. Zone heights were determined by eye using a line drawn freehand along the top of proliferating cells, the clear demarcation of cell enlargement between proliferative and hypertrophic regions, and the zone of mineralization at the base of the hypertrophic zone. The distance was measured from a point in the center of the image, and the length between each was recorded using ImageJ (NIH; Fig. 2). The total number of cells between the zone of mineralization and hypertrophy was counted by eye.

### Growth plate immunohistochemistry

Dissected tibias were fixed and embedded, and sections were cut as described earlier for metatarsals. Sections were dewaxed and rehydrated to PBS, and nonspecific antigen sites were blocked using donkey serum (1:5 dilution). Sections were incubated with a 1:100 dilution of the mouse monoclonal anti–human NKCC antibody T4(14) (affinity for both NKCC1 and NKCC2). Sections then were exposed to a 1:50 dilution of AlexaFluor 488 donkey anti-mouse (Invitrogen, Ltd.) secondary antibody for 1 hour at 37°C. Omission of the primary antibody served as a control.

Immunohistochemically stained growth plate sections were imaged using a Zeiss LSM510 upright confocal laser scanning microscope (CLSM) at low power [×10 dry objective, numerical aperture (NA) = 0.3] and high power (×63 oil immersion objective, NA = 1.4). Emitted light, following excitation of AlexaFluor 488 (excitation/emission maximum 495/519 nm) with a 488-nm argon laser, was collected through a long-pass 505-nm filter. Transmitted light also was collected to aid cell/zone identification. High-resolution (1024 × 1024) images were acquired representing a field of view 920 and 206 µm^2^ and using sequential optical *Z*-steps of either 10 or 1 µm for low- or high-power lenses, respectively. Detector sensitivity was adjusted to provide maximum contrast without pixel saturation and excessive dye bleaching. Adjacent fields of view were acquired comprising the entire depth of the growth plate.

In order to identify intracellular and cytoplasmic membrane-associated immunofluorescence in an unbiased manner, the cell membrane was visualised using high-power images obtained with transmitted light images. A line was plotted through the central axis of the middle optical section for each cell. The average fluorescent signal between the two cytoplasmic membrane segments and the central intracellular portion were recorded. To correct for variations in sectioning angle between metatarsals, the growth plate was partitioned into eight equidistant sections.(2) The top of section 1 (S_1_) was taken as the top of the proliferating zone, with the bottom of section 8 (S_8_) at the zone of mineralization. Section average intracellular and cytoplasmic membrane fluorescence was determined from all cells within each section. The fluorescence intensity was expressed as a percentage; the most intense intracellular or cytoplasmic membrane section measurement was assigned 100%, thereby normalizing images from different growth plates.

### Microarray analysis

The transcriptional analysis of cDNA by microarray used here has been described previously in detail.(11) Briefly, serial 6-µm sections of snap frozen tibia were cut using a Leica CM3050 cryostat onto Leica polyethylennaphtalae slides (PEN, Leica Microsystems, Bannockburn, IL, USA) and kept on dry ice immediately prior to laser capture microdissection (LCM). A Leica Application Solution Laser Microdissection instrument (Leica Microsystems) with either a ×10 or ×20 objective lens was used to cut freehand-traced areas. The morphology of PZC and HZC was easily identified by eye. Each growth plate fragment fell directly into an RNase-free microfuge tube containing RLT lysis buffer (Qiagen, Valencia, CA, USA). Approximately three regions of PZ and HZ were collected from each slide, with each zone harvested into separate tubes. Total RNA was extracted using the RNeasy RNA isolation kit (Qiagen). Pooled samples contained between 30 and 50 ng of RNA (RNA Pico Labchip, Agilent Technology Bioanalyzer, Santa Clara, CA, USA). After purification and quantification of mRNA,(11) 15 µg of biotinylated cRNA was hydrolyzed to 35 to 200 nucleotides, and known concentrations of positive control genes were added (50 pM Oligo B2 and 1.5, 5, 25, and 100 pM of *Escherichia coli bioB, bioC, bioD*, and *cre*). The hybridization solution was heated (45°C for 5 minutes) and centrifuged (5 minutes) before each sample was injected into an individual RAE230 2.0 GeneChip (Affymetrix, Santa Clara, CA, USA). Chips were hybridized at 45°C for 16 hours with constant rotation (60 rpm) and then were washed and stained on the Fluidics station (Affymetrix) according to the EukGE-WS2v4 protocol. Fluorescent images were acquired using the Agilent G2500A Gene Array Scanner. Affymetrix software (MicroArray Suite 5.0) was used to process the raw images into Cel images files, which were loaded into GeneSpring (Agilent Technologies, Pale Alto, CA, USA) and normalized using the robust multiarray averaging (RMA) method. Differential gene expression ratios were considered significant at the 95% confidence interval (CI).(15)

### Real-time RT-PCR

cDNA was produced from the pooled RNA samples used for the arrays by reverse transcription (RT) of 1 µg of total RNA with M-MLV reverse transcriptase and oligo-(dT)15 primers (Promega, Madison, WI, USA). Total RNA from a neonatal (3-day-old) rat kidney was similarly reverse transcribed to generate a positive control for all primer sets, which were run in duplicate. Negative controls included an RT-null control (no RT enzyme), cDNA-null for each primer set, and a template/primer null reaction.

Primers were synthesized by MWG Biosciences (Huntsville, AL, USA): 5′GTCTAAGGACCTGCCACCAA3′ (NKCC1), 5′CGGGTCGTCTAGATCCAAAA3′ (NKCC2), and 5′AGCCATGTACGTAGCCATCC3′ (β-actin, a housekeeping gene sequence, was used for normalization) and antisense primers 5′TGCTGACGATCCAGTCACTC3′, 5′ATGGACTTGGAAACGACTGG3′, and 5′CTCTCAGCTGTGGTGGTGAA3′, respectively. Prior to performing the real-time assay, the annealing temperature was optimized by gradient PCR using HotStartTaq (Qiagen). Probe specificity was assumed from visualization of a single band at the expected molecular size by agarose gel (2%) electrophoresis.

PCR was performed with Quantitect SYBR Green PCR master mix (Qiagen). The total reaction volume was 25 µL with 12.5 µL of the master mix, primers at 0.3 µM (0.075 µL), and 0.5 µL of the RT product cDNA and brought to balance with nuclease-free water. The reactions were run in duplicate for 40 cycles using an ABI Prism7000 Sequence Detection System (PE Applied Biosystems, Foster City, CA, USA). The reaction parameters were 95°C for 15 minutes (hot start), denatured at 94°C for 15 seconds, annealed at 58°C for 15 seconds, and extension/read at 72°C for 30 seconds. After the fortieth cycle, dissociation data were collected from 66.4 to 91.7°C in 0.9°C increments.

### Data analysis

Data were expressed as means ± SEM obtained from a number of individual animals (*n*). Differences between means were determined by paired Student's *t* test, with *p* < .05, *p* < .01, and *p* < .001 considered to be significant, highly significant, and extremely significant, respectively. Statistical analysis was performed using SigmaStat (Systat, Chicago, IL, USA).

## Results

### Bumetanide bone rudiment culture

Duplicate paired metatarsal and metacarpal bones from P_7_ rat pups exhibited a dose-dependent inhibition of bone lengthening by bumetanide (Fig. 1). Bone lengthening was significantly reduced with bumetanide concentrations of 12.5, 100, and 200 µM (*p* < .05, *t* test) compared with controls. A curve fit of the bumetanide dose-response data was best fitted by a three-parameter hyperbolic decay curve (*r*^2^ = 0.9656, correlation coefficient), with predicted maximal longitudinal growth of 1.725% as [bumetanide] → ∞ compared with growth in control bones of 3.69% after 24 hours of incubation. Owing to the differing growth rates of metacarpals and metatarsals,(16) further experiments were performed using faster-growing metatarsals alone (Fig. 1, *inset*). Control metatarsals increased in length by approximately 6% after 24 hours in culture. When incubated with 100 µM bumetanide, bone lengthening was significantly reduced (*p* < .01, Students *t* test) by approximately 35%.

Measurement of the growth plate of the metatarsals, from uppermost PZC to the zone of mineralization, is shown in Fig. 2. There was no overall difference in total growth plate length between control and bumetanide-treated samples (Table 1), although these measurements were associated with a large SEM. However, there was a significant reduction (*p* < .05, Student's paired *t* test) in the hypertrophic zone of approximately 50 µm. When the data were normalized with the HZ expressed as a percentage of the total growth plate height, a highly significant reduction of nearly 50% was observed in the bumetanide-exposed specimens (Table 1, *p* < .01, Student's *t* test).

**Table 1:** Reduction in Height of the Growth Plate Hypertrophic Zone by Bumetanide

Zone	Control	Bumetanide 100 µM	*p* Value
Total growth plate (µm)	636 ± 150	689 ± 130	.655
Late HZ (µm)	204 ± 25.6	151 ± 15.0	.015*
HZ (% of total)	33.8 ± 1.57	22.7 ± 0.814	.008**

*Note*: Growth plate measurements of three P_7_ rat metatarsals (proximal and distal) cultured for 24 hours in the absence/presence of 100 µM bumetanide. Bones were fixed, prepared, and visualized (Fig. 2) as described in “Materials and Methods.” The average height of growth plates was not significantly different (paired Student's *t* test) between control and bumetanide-treated bones, whereas there was a significant (*p < .05, paired Student's *t* test) decrease by approximately 50 µm in the late hypertrophic zone (HZ). When normalized and presented as a percentage of total growth plate height, the reduction in growth was highly significant (**p < .01, Student's paired *t* test). Data shown as means ± SEM.

HZ cell counts of each micrograph were almost identical (*p* = .937, Student's *t* test), with 193 ± 11.3 cells for the control group and 192 ± 13.6 cells for the bumetanide-treated group. When corrected for the section area, no significant difference (*p* = .953, Student's *t* test) existed between the cell densities of control and bumetanide-treated groups (1400 ± 125 and 1377 ± 350 cells/mm^2^, respectively), showing that the number of HZCs did not change with exposure to bumetanide.

### Fluorescence immunohistochemistry

**Fig. 3:**
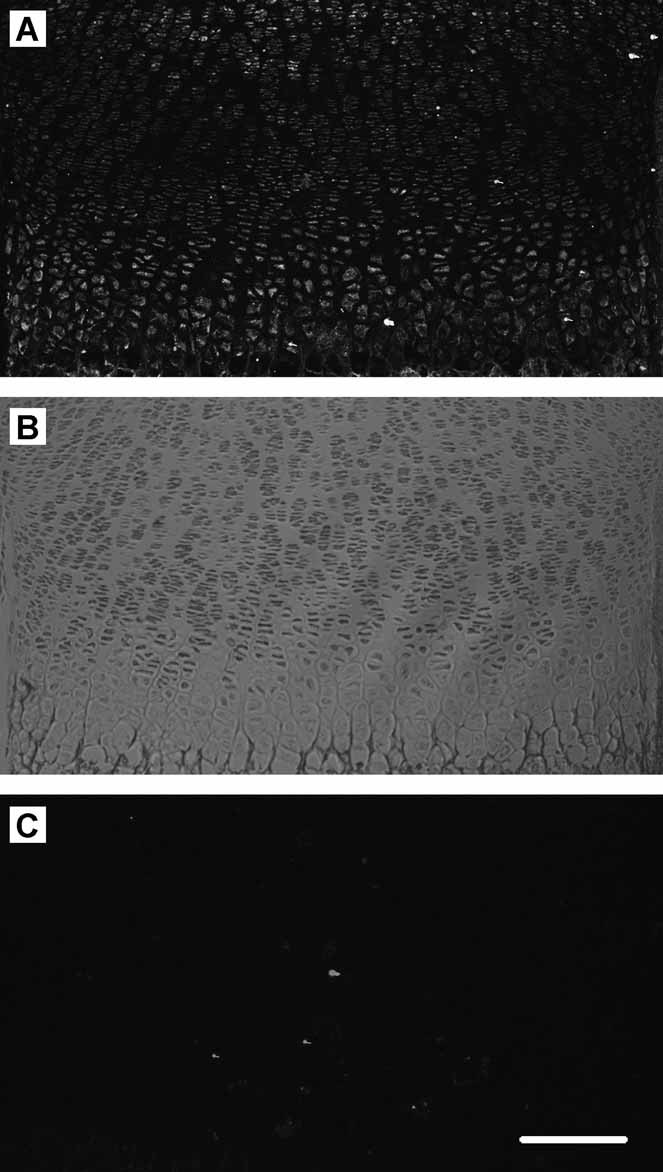
Growth plate NKCC distribution. Low-power (×10 objective) confocal microscope images (projected) of proximal tibial growth plate stained for NKCC (T4 antibody). Rat proximal tibias were removed and fixed, and transverse sections were prepared for immunohistochemistry as described in “Materials and Methods.” (*A*) AlexaFluor 488 secondary antibody fluorescence, (*B*) transmitted light image of the same field of view, and (*C*) as for panel *A* but with primary antibody omitted. The proliferative zone showed some fluorescence that appeared to reduce to the upper edge of the HZ. Cells in the HZ exhibited strong fluorescence, which even at this low magnification appeared to be localized to the plasma membrane. Scale bar = 200 µm.

Immunohistochemistry was used to determine both the tissue and cellular localization of NKCC in the mammalian growth plate. The T4 monoclonal anti-NKCC antibody exhibited strong staining for chondrocytes throughout proximal tibial growth plate cartilage from P_7_ animals, although the pattern of fluorescence staining was heterogeneous. Low-power (×10 objective lens) CLSM images of NKCC immunohistochemistry (Fig. 3) showed the distribution of staining along the growth plate. It can be seen that the fluorescent signal was strong in the early proliferative zone, decreasing through the late proliferative and early hypertrophic regions, before increasing again in the middle to late hypertrophic zones. This observation was consistent across proximal tibial growth plates of all seven animals.

**Fig. 4:**
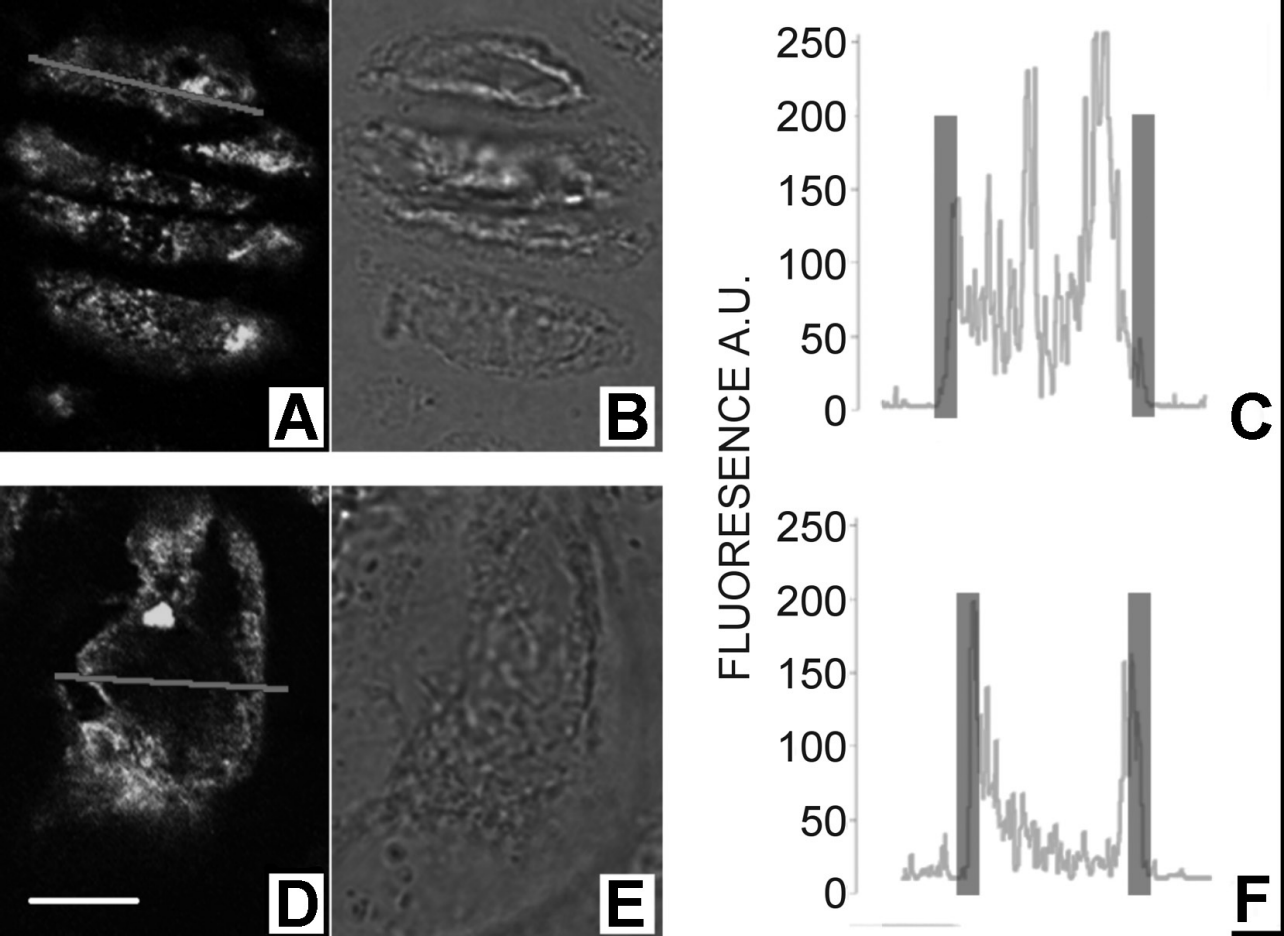
Growth plate chondrocyte NKCC localization in typical proliferative (*A–C*) and hypertrophic (*D–F*) zone chondrocytes. Rat proximal tibias were prepared for and subjected to immunohistochemical procedures, and then images were acquired as described in “Materials and Methods.” NKCC immunofluorescence from a single optical section is shown in panels *A* and *D*, with the corresponding transmitted light images (*B*) and (*E*) for PZCs and HZCs, respectively. The intensity profile along a line drawn through the center of a cell (gray line in *A* and *D*) is shown (*C*, *F*). The overlaid transparent vertical bars in *C* and *F* indicate the approximate position of the cytoplasmic membrane determined from the transmitted light image (*B*, *E*). Fluorescence intensity is in arbitrary units (AUs). For all panels, scale bar = 5 µm.

**Fig. 5:**
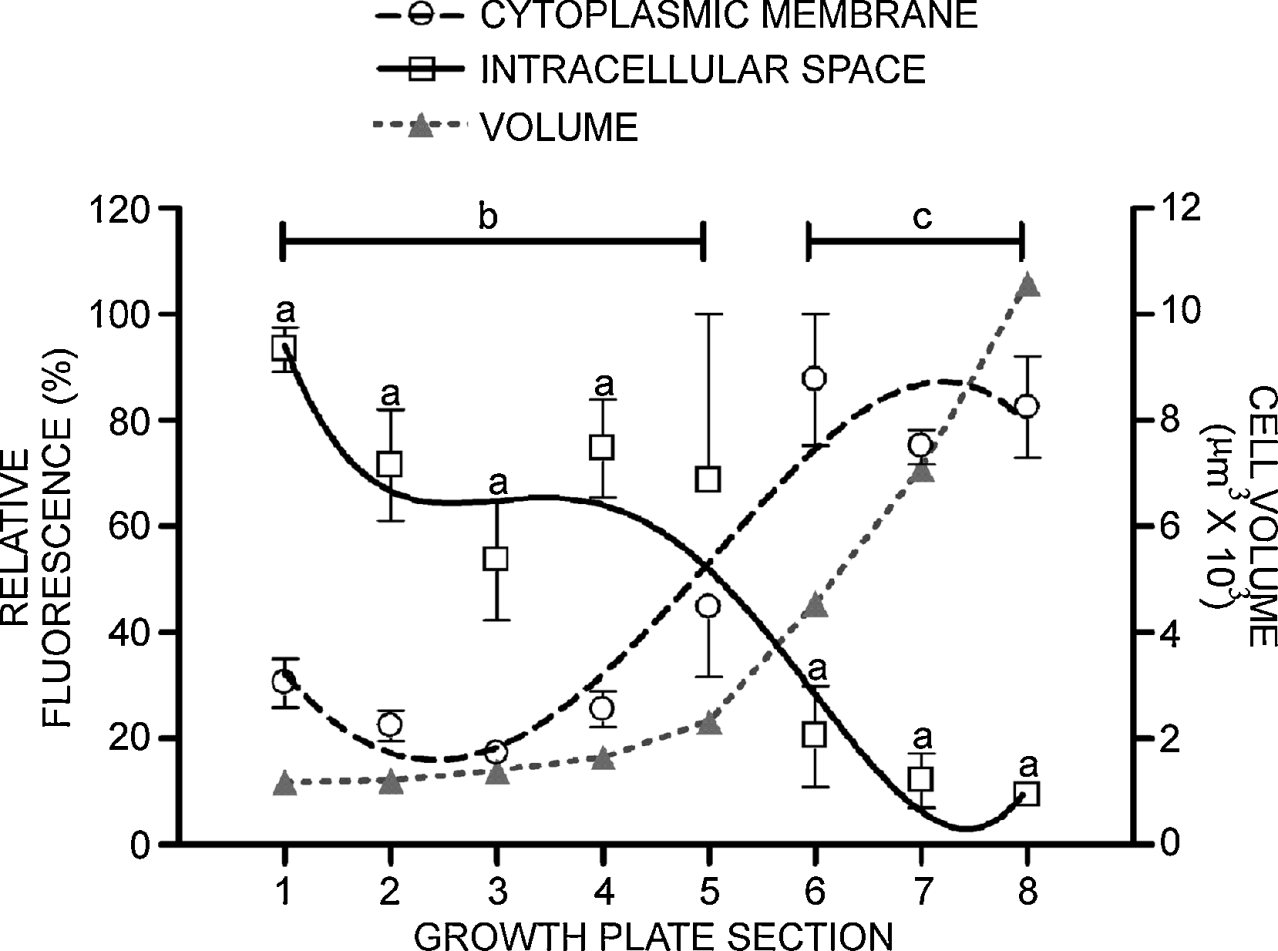
Quantification of cellular NKCC distribution along the growth plate. The fluorescence associated with the plasma membrane or intracellular space was obtained from images of cells along the entire length of the growth plate (see “Materials and Methods” and Fig. 4). Owing to the variability in section position, angle, and inherent differences in growth plate dimensions, cell positions within the growth plate were expressed in eight equidistant sections; sections 1 to 3 PZC and 5 to 8 HZC. Significant differences (^a^*p* < .05, Student's *t* test) between the fluorescent signal of the intracellular space (*solid line*, □) and membrane-associated fluorescence (*broken line*, ○). Similarly, significant differences (*p* < .05, Student's *t* test) between PZC and HZC intracellular fluorescence (*b*) and between PZC and HZC membrane-associated fluorescence (*c*) are shown. An overlay (*gray line*, ▵) of cell volumes (*right axis*) has been included to highlight the appearance of NKCC in the plasma membrane immediately preceding a volume increase (data replotted from ref. (2)). Data points are for the mean of a minimum of four animals (421 cells) with bars denoting SEM.

High-power (×63 objective lens) images allowed the observation of cellular localization, which differed as cells transitioned from early PZCs to late HZCs. Figure 4*A, D* shows typical PZCs and an HZC with a predominantly intracellular and cytoplasmic membrane-associated fluorescence, respectively. Quantification of immunofluorescence (Fig. 5) confirmed that qualitative assessment of intracellular fluorescence was highest in the PZC, with S_1_ to S_5_ being significantly higher than S_6_ to S_8_ (*p* < .05, paired Student's *t* test). In contrast, cytoplasmic membrane–associated fluorescence was lowest in S_1_ to S_4_ PZCs, increasing significantly in S_6_ to S_8_ (*p* < .05, paired Student's *t* test). Within each growth plate section S_1_ through S_8_, there was a significant difference (*p* < .05, paired Student's *t* test) between intracellular and cytoplasmic membrane–associated fluorescence with the exception of cells from S_5_.

### Gene microarray and RT-PCR analysis

Good-quality antibodies raised against the different isoforms of NKCC (1 and 2) were not available for immunohistochemistry, and hence an alternative approach of gene microarray analysis was required to confirm the presence of NKCC1 over NKCC2. Of the 161 genes examined as part of a wider study into membrane-transport proteins, the majority (approximately 90%) showed low expression levels [<10 arbitrary expression units (AEUs)]. Indeed, the average raw expression levels for the 161 transport-protein genes across both PZCs and HZCs at all time points studied was 8.997 (ranging from 3.15 to 308 AEUs). As expected, the kidney-specific *NKCC2* gene showed background (<10 AEUs) expression levels, whereas the ubiquitous *NKCC1* gene expression levels were above background (Table 2). The positive control proteins for PZC and HZC (procollagen type II and matrix metalloprotease 13 [MMP13], respectively) exhibited high expression levels.

**Table 2:** Microarray mRNA Expression of *NKCC1*, *NKCC2*, *MMP13*, and *Col II* in Rat Growth Plate Chondrocytes

	Expression (AEUs)					
	
	Day 49		Day 53		Day 58	
			
Gene	PZC	HZC	PZC	HZC	PZC	HZC
*NKCC1*	19.4	56.9	16.1	14.1	79.8	189.4
*NKCC2*	3.21	4.23	3.31	4.31	4.36	3.15
*MMP13*	5.72	1019	9.34	676	39.6	5241
*Col II*	6999	5762	4838	2275	8478	6617

*Note*: Mean raw levels (AEUs) from five animals each, shown for kidney-specific NKCC2 and ubiquitous NKCC1 transport proteins and the HZC and PZC markers MMP13 and procollagen type II, respectively. The NKCC2 isoform showed no expression above background levels (<10 AEUs), whereas NKCC1 exhibited expression well above average for the 161 transport protein/carrier genes analyzed (∼9.0 AEUs). The high expression levels of the standard markers MMP13 and procollagen type II highlight the low levels associated with NKCC1 and NKCC2 isoforms.

Statistical significance was determined at the 95% confidence interval (CI) with a two-tailed cutoff of 1.96 SD. Log_2_ values are shown in Table 3 for the ratios (HZC:PZC) of NKCC1, NKCC2, MMP13, and procollagen type II on rats aged 49, 53, and 58 days. With log_2_ values that exceed the 95% CI, it would be expected that there would be a less than 5% risk of false-positive results. For MMP13, since the log_2_ ratios exceed the 95% CI by at least 8 SDs in one time point, we should expect no false-positive results. However, of the NKCC transport proteins, only NKCC1 expression on day 49 exceeded the 95% limit. A twofold HZC:PZC ratio was considered relevant(13); as expected, MMP13 exhibited a large increase between PZCs and HZCs (Table 3), and NKCC1 showed a greater than twofold change on days 49 and 58. There was little change from unity for NKCC2 expression between PZCs and HZCs, near background in both zones across the three animal ages studied.

**Table 3:** Microarray Analysis of Growth Plate *NKCC1*, *NKCC2*, *MMP13*, and *Pro-Col II*

	Log 2 ratio (HZ/PZ)			Ratio (HZ/PZ) fold change		
		
Gene	Day 49	Day 53	Day 58	Day 49	Day 53	Day 58
*NKCC1*	1.55	−0.20	1.25	2.93	−1.15	2.37
*NKCC2*	0.40	0.38	−0.47	1.32	1.30	−1.38
*MMP13*	7.48	6.18	7.05	178.3	72.5	132.3
*Col II*	−0.28	−1.09	−0.36	0.823	0.470	0.780

*Note*: Log_2_ HZC/PZC ratios and expression ratio fold changes (HPZ/PZC) from microarray analysis (see “Material and Methods”) of *NKCC1* and *NKCC2* and the HZC and PZC positive controls *MMP13* and *Procollagen Type II*, respectively. Only the log_2_ of *NKCC1* ratio on day 49 (double-scored boundary) was greater than the 95% confidence level (1.20), whereas both day 49 and day 58 exhibited greater than twofold changes in the HZC/PZC ratios. All *MMP13* data showed relevant increases in expression between PZC and HZC and *Procollagen Type II* on day 53.

Owing to the temporal variation in NKCC1 expression by microarray, confirmation was obtained by RT-PCR (Table 4). Log_2_ ratios and fold increase in expression between PZ and HZ were identical to those obtained by microarray; indeed, plotting both sets of results by linear regression produced a coefficient of determination (*r*^2^) of 0.986. Similar analysis of NKCC2 revealed a poor correlation (*r*^2^ = 0.116), which was more indicative of the levels below threshold associated with NKCC2.

**Table 4:** Real-Time RT-PCR Analysis of *NKCC1* and *NKCC2*

	Log 2 ratio (HZ/PZ)			Ratio (HZ/PZ) fold change		
		
Gene	Day 49	Day 53	Day 58	Day 49	Day 53	Day 58
*NKCC1*	1.7	−0.175	1.12	3.25	−1.13	2.17
*NKCC2*	0.305	0.8	0.305	1.24	1.74	1.24

*Note*: Log_2_ HZC/PZC ratios and expression ratio fold changes (HPZ/PZC) from real-time RT-PCR (see “Material and Methods”) of *NKCC1* and *NKCC2*. The results for *NKCC1* mimic those obtained in the microarray analysis, with both day 49 and day 58 animals exhibiting greater than twofold changes in the HZC/PZC ratios. The coefficient of determination (*r*^2^) for the linear regression between RT-PCR and microarray analysis was 0.986 for *NKCC1* and 0.116 for *NKCC2*.

### DIDS bone rudiment culture

**Fig. 6:**
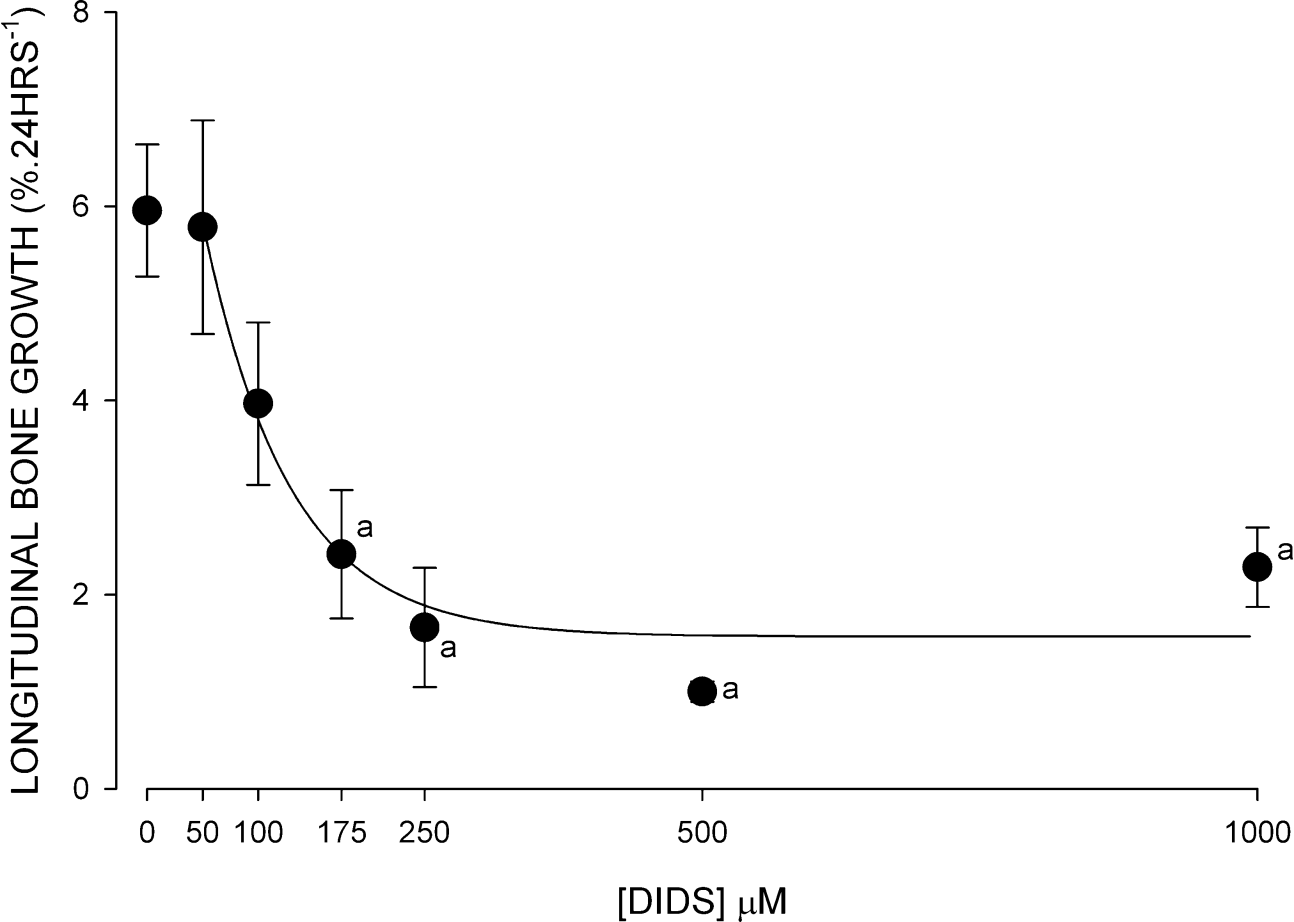
The effect of the AE inhibitor DIDS (in the presence of 100 µM of bumetanide) on rat metatarsal/metacarpal bone rudiment growth. Each data point represents a metatarsal and metacarpal from of each of at least four animals, cultured for 24 hours, and measured as described in “Materials and Methods.” There was a dose-dependent relationship of DIDS inhibition, significantly reduced (^a^*p* < .05, *t* test) from control at DIDS concentrations of 175 µM or greater with maximal inhibition at 500 µM. Data points are for the mean of a minimum of four animals with bars denoting SEM.

Metatarsal and metacarpal bones from P_7_ rat pups exhibited a dose-dependent inhibition of bone lengthening by DIDS in the presence of 100 µM bumetanide (Fig. 6). Bone lengthening was significantly further reduced by DIDS concentrations of 175 µM to 1 mM (*p* < .05, *t* test) compared with 100 µM of bumetanide alone. Rudiment culture for 24 hours in the presence of 500 µM of DIDS resulted in a maximal inhibition of approximately 80% over and above bumetanide alone (*p* < .001, *t* test).

## Discussion

This study set out to examine the involvement of NKCC1 in the coordinated volume increase of growth plate HZCs. Using a variety of techniques, we provide evidence that implicates NKCC1 in growth plate chondrocyte hypertrophy and hence longitudinal bone growth. The optimization of different techniques required the use of different bones from differently aged animals. Measurable increases in bone length were possible only with small bones from young animals, but the growth plate chondrocytes of these bones do not withstand crushing injury caused by the bisection required for cell volume determination by CLSM. Hence larger tibias were used from P_7_ animals to directly compare the relationship between cell volume and NKCC immunohistochemistry. Although the metatarsals, metacarpals, and tibias used for immunohistochemistry were from age-matched animals (P_7_), their relatively small growth plates make extracting sufficient mRNA for microarray analysis technically demanding. Hence older animals with larger growth plates were used, a temporal effect partially checked by examining tibias from P_49_, P_53_, and P_58_ animals.

Microarray analysis of raw data (Table 2) indicated a substantial increase for P_49_ and P_58_ animals but no increase at P_53_. This may be an indication of temporal changes in NKCC activity, but when compared with MMP13 and Pro-Col II data, it is more likely due to a general suppression of hypertrophy-related genes in this experimental set. Further analysis revealed that while both P_49_ and P_58_ animals showed a greater than twofold increase in NKCC1 expression between PZCs and HZCs, only the data from P_49_ animals exceeded the 95% confidence limit. Some confusion therefore exists with the microarray data; however, subsequent RT-PCR analysis of the samples provided confirmation of these results. It must be remembered that the primary role for this technique was to confirm the presence of NKCC1 over NKCC2, albeit not from P_7_ rats. The negligible signal for *NKCC2* mRNA gives confidence that we were able to associate T4 antibody immunohistochemistry to NKCC1 alone.

The T4 monoclonal antibody used for NKCC immunohistochemistry has been used previously in mammalian tissue for successful tissue immunolocalization of NKCC in rat hippocampus,(17) gerbil inner ear,(18) rabbit parotid and kidney,(19) and human airway smooth muscle.(20) Similarly, T4 resulted in clear CLSM images of NKCC distribution in growth plate sections (Figs. 3 and 4). Quantification of NKCC immunohistochemistry cellular localization (Fig. 5) showed that NKCC was present along the entire growth plate, but with a change in immunolocalization of NKCC from intracellular regions to the cytoplasmic membrane with transition from PZCs to HZCs. Localization of NKCC to the cell membrane is essential for its involvement in HZC volume increase.

While not of primary concern to the role of HZC volume increase, the intracellular localization of NKCC in PZCs is intriguing. PZC NKCC appeared to be adjacent to the nucleus, suggesting an association with endoplasmic reticulum and/or the Golgi apparatus. This could represent the synthesis of NKCC pools ready for translocation to the plasma membrane for future cell volume increases. Such a redistribution of the cotransporter to the cell surface is a mechanism for NKCC upregulation in other cell types.(21) Similar differences in NKCC distribution between PZCs and HZCs are also observed with human cytomegalovirus infection of a fibroblast cell line.(22) Infection inhibits translocation of NKCC to the plasma membrane, resulting in similar perinuclear distribution as shown here, with abolition of cell NKCC activity.

The tissue distribution and cellular localization of NKCC were consistent with its role in HZC volume increase, but it is the bumetanide inhibition of NKCC that provides direct evidence for a role in bone lengthening. The precise bumetanide dose required to inhibit bone lengthening is difficult to determine because in situ the cells would experience less than that added to the culture medium, but it may be similar to the bumetanide concentration required for maximal inhibition of NKCC in isolated cells (≤20 µM). Conversely, the maximum concentration of bumetanide used (200 µM) would ensure that in situ chondrocytes were not exposed to levels associated with nonspecific inhibition of bicarbonate exchanger,(12) chloride channels,(13) and potassium chloride cotransport.(14)

Approximately 80% of bone lengthening has been attributed to HZC volume increase.(1) Therefore, the inhibition of metatarsal longitudinal growth by bumetanide accounts for less than half, if one assumes that NKCC inhibition by bumetanide was limited to HZCs. PZC cell division contributes to approximately 10% of bone lengthening(1) and may be inhibited by bumetanide.(23–25) However, NKCC distribution suggests only low levels of NKCC in the cytoplasmic membrane of PZCs, and histologic analysis (Fig. 2) of bumetanide-treated metatarsals does suggest that inhibition was restricted to HZCs.

The partial inhibition of bone lengthening by bumetanide would suggest the presence of other mechanisms to drive HZC volume increase. Inorganic ion transporters also known to be involved in RVI may be implicated, such as increased activity of sodium hydrogen exchange (NHE), anion exchange (AE), and sodium chloride cotransport (NCC) or decreased activity of ion-transport proteins known to bring about a decrease in cell volume, including potassium-driven transporters and the Na^+^/K^+^ pump.(6) Of all these membrane proteins, microarray analysis highlighted AE3 (Slc4a3) alone as changing expression between proliferative and hypertrophic zones, with an approximately 14-fold increase (absolute expression values of 217 to 3122) compared with no change for AE1 (Slc4a1; 255 to 216, −1.18-fold change) or AE2 (Slc4a2; 105 to 109, 1.04-fold change). Metatarsal culture experiments in the presence of 100 µM bumetanide and the AE inhibitor DIDS (0 to 1 mM) resulted in a dose-dependent inhibition of bone lengthening greater than bumetanide alone (Fig. 6), inhibiting bone lengthening by a maximum of 80% compared with control bones.

Growth plate chondrocyte AE3 would appear to be a candidate for driving the major portion of growth plate chondrocyte volume increase; however, a number of concerns with this hypothesis exist. First, DIDS is a relatively nonspecific inhibitor of carrier-mediated anion transport.(26) Indeed, in HZ growth plate chondrocytes, DIDS has been shown to completely abolish diastrophic dysplasia sulfate transport (DTDST)–mediated sulfate uptake critical for proteoglycan synthesis.(27) Second, AE must be coupled with other transport proteins to elicit a volume-regulatory increase, typically sodium hydrogen exchange,(6) but these transport proteins show no increase in mRNA expression between PZCs and HZCs. Finally, we cannot be certain that DIDS inhibition of anion transport does not exert its inhibition of bone lengthening through modulation of intracellular pH. Unlike NKCC, with its specific inhibitors (ie, bumetanide and furosemide), the role of AE3 cannot be ascertained because it has no analogous specific inhibitors, but its role clearly warrants further investigation.

The putative presence of transporters other than NKCC1 and/or redundancy in the system to drive HZC volume increase may explain the reduced size of *NKCC1* knockout mice over the first 2 weeks, a period of maximal growth rate,(16) but their ability to recover to the size of wild-type animals as they reach maturity.(28) This ability of *NKCC1* knockouts to recover may explain why there are limited reports of skeletal growth retardation in children exposed to loop diuretics (eg, bumetanide and furosemide). Reduced bone growth also may go unreported owing to the conditions that required prescription of loop diuretics. Pediatric furosemide therapy is used in the treatment of chronic lung disease, nephritic syndrome, renal failure, congestive heart failure, and hydrocephalus fluid shunts (see ref. (29)), conditions that might be expected to result in poor growth outcome and therefore not subsequently associated with loop diuretics.

A study of four children prescribed the NKCC inhibitor furosemide(30) described bone demineralization, but no mention was made of reduced longitudinal bone growth. However, anecdotal clinical reports led to a study by Koo and colleagues,(31) who subjected 4-day-old rats to furosemide for 24 days and observed a significant reduction in tibial length that was not associated with abnormal calcium, sodium, magnesium, potassium, or parathyroid hormone in plasma or bone. This suggested that bone growth and not subsequent demineralization was responsible. Although no explanation was provided for the bone growth retardation observed, the present findings could implicate direct NKCC1 inhibition of HZCs in vivo.

If NKCC plays the major role of HZC volume increase, it would be expected to be under the same hormonal control that regulates bone growth. NKCC has been shown to be regulated by hormones in other tissues.(32) Future work is required to elucidate this, as well as to study NKCC1 regulation through control of protein synthesis, translocation to the cytoplasmic membrane, and activation through phosphorylation/dephosphorylation events(22,33,34) to provide an insight into the control of HZC volume increase mediated by NKCC1. The regulated activation of NKCC1 within the mammalian growth plate also could provide an ideal model for the study of general NKCC1 physiology.

In summary, we hypothesised that NKCC activity may play a role in the coordinated volume increase seen in HZCs of the growth plate. Using multiple methodologies, we have provided evidence for the role of NKCC1 in growth plate HZC volume increase: the inhibition of bone lengthening (through reduction in HZ height) with the NKCC-specific inhibitor bumetanide, appropriate cellular localization of NKCC in HZCs to the cytoplasmic membrane, and an increase of *NKCC1* mRNA in HZCs with negligible *NKCC2* signal. The involvement of NKCC in bone lengthening warrants further investigation, with special attention to the detrimental therapeutic effects possible with NKCC1 inhibitors (loop diuretics) in children.
